# Trapped in Statelessness: Rohingya Refugees in Bangladesh

**DOI:** 10.3390/ijerph14080942

**Published:** 2017-08-21

**Authors:** Abul Hasnat Milton, Mijanur Rahman, Sumaira Hussain, Charulata Jindal, Sushmita Choudhury, Shahnaz Akter, Shahana Ferdousi, Tafzila Akter Mouly, John Hall, Jimmy T. Efird

**Affiliations:** 1 Centre for Clinical Epidemiology and Biostatistics (CCEB), School of Medicine and Public Health, The University of Newcastle (UON), Newcastle 2308, Australia; milton.hasnat@newcastle.edu.au (A.H.M.); mdmijanur.rahman@uon.edu.au (M.R.); charulata.jindal@newcastle.edu.au (C.J.); 2 Mercycorps, Pak Palace, Murree Road, Rawal Chowk, Islamabad 45510, Pakistan ; sumaira.87@gmail.com; 3 Newcastle Law School, Faculty of Business and Law, The University of Newcastle (UON); Newcastle 2308, Australia; sushmita.choudhury@newcastle.edu.au; 4 School of Medicine and Public Health, the University of Newcastle (UON), Newcastle 2308, Australia; shahnaz.akter@uon.edu.au; 5 WentWest Limited, Western Sydney Primary Health Network (WSPHN), Sydney 2148, Australia; shahana.Ferdousi@wentwest.com.au; 6 Centre for Health and Development (CHAD), Dhaka 1219, Bangladesh; tafzila.mouly@yahoo.com.au; 7 School of Public Health and Community Medicine, University of New South Wales (UNSW), Sydney 2052, Australia; john.hall@unsw.edu.au; 8 Center for Health Disparities (CHD), Brody School of Medicine, East Carolina University (ECU), Greenville, NC 27834, USA

**Keywords:** Rohingya refugee, statelessness, Bangladesh, Myanmar

## Abstract

The Rohingya people are one of the most ill-treated and persecuted refugee groups in the world, having lived in a realm of statelessness for over six generations, and who are still doing so. In recent years, more than 500,000 Rohingyas fled from Myanmar (Burma) to neighboring countries. This article addresses the Rohingya refugee crisis in Bangladesh, with special emphasis on the living conditions of this vulnerable population. We reviewed several documents on Rohingya refugees, visited a registered refugee camp (Teknaf), collected case reports, and conducted a series of meetings with stakeholders in the Cox’s Bazar district of Bangladesh. A total of 33,131 registered Rohingya refugees are living in two registered camps in Cox’s Bazar, and up to 80,000 additional refugees are housed in nearby makeshift camps. Overall, the living conditions of Rohingya refugees inside the overcrowded camps remain dismal. Mental health is poor, proper hygiene conditions are lacking, malnutrition is endemic, and physical/sexual abuse is high. A concerted diplomatic effort involving Bangladesh and Myanmar, and international mediators such as the Organization of Islamic Countries and the United Nations, is urgently required to effectively address this complex situation.

## 1. Introduction

The global refugee crisis is an ongoing concern, with the United Nations High Commissioner for Refugees (UNHCR) reporting a sharp increase in forcibly displaced populations from 59.5 million in 2014 to 65.3 million in 2015 [1]. Under international law, a refugee is defined as someone who lives outside his or her country of nationality or usual residence, who is able to show a well-founded fear of persecution on specific grounds, and who lacks protection from their country [2]. The definition of displaced individuals as a result of persecution overlaps considerably with that of stateless persons, who are described as individuals not considered as a national by any state [3]. Accessing basic rights such as healthcare, employment, education and freedom of movement is often impossible for stateless people [4]. Lack, denial or loss of nationality underlies the exclusion of affected individuals from membership in the community, to the point of instigating discrimination and oppression in certain cases. There are approximately 10 million stateless people [4], and approximately 1.5 million people who are refugees in addition to being stateless [5].

Rohingya in Myanmar are one of the most persecuted minorities in the world. The majority are not considered to be citizens by the Myanmar Government, and live in a condition of statelessness. Rohingya are a Muslim ethnic minority situated primarily in Myanmar’s western Rakhine State and are estimated at 1 million people [6]. They have been fleeing Myanmar in large numbers, often to nearby developing countries—particularly Bangladesh, Malaysia and Thailand—to avoid conflict and persecution [7]. Correspondingly, the refugee crisis in Bangladesh has reached critical levels, with the number of unregistered Rohingya refugees estimated to range from 200,000 to 500,000 people [8].

The plight of the Rohingya dates back two centuries. Rohingyas’ history can be described in three categories: precolonial, colonial and postcolonial. In precolonial times, the independent kingdom of Arakan (currently known as the Rakhine state), was populated by Muslim Arabic sailors from 788 to 810 AD, and afterwards by Bengalis from the fifteenth to the seventeenth centuries [9]. During precolonial times, the Rohingyas and Arakanese (the remainder of the population in Arakan) lived in harmony. This changed after colonization by the British following the first Anglo-Burmese war in 1825. The rift deepened during the Second World War, when the Rohingyas declared their loyalty to the British, while the Arakanese sided with the Japanese [10]. During the Japanese occupation of Burma (including Arakan), the Rohingya population was targeted jointly by both the communalist (Buddhist) Rakhine and the Burma Independence Army, killing 100,000 Rohingya and exiling a further 50,000 towards the border to East Bengal [9,11]. After Burma received independence in 1948, the anti-Rohingya campaign persisted, marked by discrimination and denial of their citizenship rights. Around this period between 1940 and 1947, Buddhist fundamentalist extremism was on the rise.

Eventually, in 1978, the amassed anti-Rohingya sentiment culminated in the military junta operation to purge Burma of illegal inhabitants, which comprised harassment, violence and arrest. This led to the flight of 250,000 Rohingyas to Bangladesh [11]. Faced by pressure from the international community, a repatriation agreement was struck with Bangladesh the following year, resulting in the majority of Rohingyas being returned to Burma. However, just three years later, Burma passed the 1982 Citizenship Law that denied citizenship to Rohingyas, decreeing an estimated 800,000 Rohingyas in North Rakhine stateless. Rohingyas are not recognized as a national race by the Burmese government, even if there is evidence that they were born in the country, instead identifying them as “Bengali” illegal immigrants [12,13]. During military junta rule in 1988, the State Law and Order Restoration Council (SLORC) established a number of new military cantonments in the Rakhine state, focusing on the north where Muslims were situated. Land was forcefully taken from the Muslim inhabitants without compensation, so Rohingyas became ‘homeless’ in addition to ‘stateless’. Branded as illegal residents, they experienced basic human rights violations including denial of access to education, healthcare, employment, freedom of movement, religion and even limited rights to get married or have children [1,13,14]. The ongoing anti-Rohingya campaign and extreme circumstances resulted in a persistent exodus of Rohingya to safer neighboring countries, where they reside as stateless refugees.

Bangladesh has been the preferred destination for the majority of asylum-seeking Rohingyas, because of the initial recognition of their humanitarian needs along with close proximity and matching religion. In 1991–1992, there was another influx of around 250,000 Rohingya refugees to Bangladesh, owing to military crackdown in Myanmar following the failed democratic election in 1990. Although the majority of refugees were repatriated to Northern Myanmar during the following decade, many of these sought their way back to Bangladesh [13]. After 1992, Rohingyas entering Bangladesh were not officially recognized as refugees by the Government of Bangladesh (GOB). However, large numbers of Rohingyas arrived once again in Bangladesh during late 2016, following a massacre in Myanmar where hundreds of Rohingyas were murdered, faced sexual violence and thousands of houses were burned down.

With more than twenty years of continuous camp settlements, the current Rohingya refugee situation in Bangladesh has become one of the most protracted in the world. In the absence of a specific refugee policy in Bangladesh and politicization of the refugee situation, integration of Rohingyas has always been a challenge. Recently in 2014, GOB announced its national policy for managing the Myanmar refugees, which is comprised of five elements: (i) preparation of a list of unregistered refugees; (ii) provision of temporary basic humanitarian relief; (iii) strengthening of border management; (iv) diplomatic initiatives with the government of Myanmar; and (v) increasing national level coordination. There are currently two government-led refugee camps for Rohingyas in the Cox’s Bazar area. However, socioeconomic conditions of the host communities in Cox’s Bazar, one of Bangladesh’s poorest districts, has further complicated finding a durable solution for the Rohingyas in the area. Whether living in camp or non-camp areas, the Rohingya refugees have been subject to miserable living conditions marked by inadequate access to basic needs, exposure to violence, restricted movement, local hostility, and various forms of discrimination. In this review article, we focus on the current situation of Rohingya refugees in Bangladesh, with special emphasis on living conditions.

## 2. Materials and Methods

For this article, we reviewed 18 documents pertaining to the Rohingya refugee crisis [1,2,3,4,5,6,7,8,9,10,11,12,13,14,15,16,17,18], visited a registered refugee camp located in Teknaf, and conducted 20 case studies. Additionally, meetings were held with representatives from various key stakeholder groups including (1) the Refugee Relief and Repatriation Commission (RRRC); (2) UNHCR; and (3) Non-Governmental Organization (NGO) Forum for Public Health. Written permission and consensus were obtained from RRRC and UNHCR for the data presented in Table 1, Table 2, Table 3 and Table 4. RRRC granted visitation rights and approval for the refugee camp interviews.

Rohingya is a generic term for the Muslim inhabitants of Arakan, the border region of Myanmar’s western coast that was officially designated as the Rakhine state in 1989. Presently, concern is greatest for the population from three northern regions of the Rakhine State: Maungdaw, Buthidaung and Rathendaung, which are currently facing conflict. As a result, approximately 400,000 to 500,000 Rohingyas have left Myanmar to take refuge in Bangladesh [2].

To provide shelter to the Rohingya refugees, GOB constructed twenty camps in 1992. Currently, there are only two camps for documented Rohingya refugees—“Kutupalong” in Ukhia (sub-district) and “Nayapara” in Teknaf (sub-district)—from the Cox’s Bazar district located on the Bangladesh’s southeastern coast. There are several makeshift camps in the surrounding area that house unregistered Rohingya refugees, for example Leda Camp. Without registration, however, these camps are not conferred legal protection. As a result, refugees there are at higher risk of violence, physical and sexual abuse, arrest, and detention. Furthermore, registered refugees receive support in terms of shelter, food assistance, education, water, sanitation, health and nutrition from GOB, UNHCR, international NGOs and local NGOs, whereas unregistered refugees living in makeshift camps have limited access to shelter, water, sanitation and health services, and are not entitled to food assistance by mandate of GOB [15].

## 3. Results

Of the 400,000–500,000 Rohingyas living in Bangladesh, 33,131 are registered as refugees living in two camps (Ukhiya and Teknaf). However, there are 63,000–80,000 undocumented refugees living in nearby makeshift camps.

There are schools and medical facilities (in-patient and out-patient) available for registered refugees in the camp (Table 1). However, access to education remains partial and ad hoc, with no entitlement to education for non-camp Rohingya children. Table 2 illustrates the distribution of the registered Rohingya refugee population based on age and sex. Children comprise one-fourth of the camp population, and most were born in the camp.

The morbidity and mortality status of the registered Rohingya refugees are presented in Table 3. Both camps have clinics for providing a basic level of healthcare free of charge, which includes limited referrals to specialists in the Cox’s Bazar and Chittagong districts. Overall mental health conditions, as well as epilepsy/seizures, are a concern in both camps. Psychotic disorders are also prevalent, although precise data are unavailable for Nayapara.

Table 4 presents information on access to healthcare, water and sanitation. Access to antenatal care, antenatal tetanus vaccination and birth attendance is a key focus of the clinics. However, access to clean water remains a problem, particularly in Nayapara, owing to shortage of groundwater. In the camps, rainwater is channeled into a basin, cleaned and then pumped to distribution points twice daily for two hours.

### Case Studies

In 2012, one of the co-authors (SC), a member of the Bangladesh National Human Rights Commission’s (NHRC) fact-finding team, conducted the Rohingya refugee case studies on-site, each lasting approximately 90 min. Participants were equally distributed by sex. These observations revealed important findings such as unavailability of education facilities for undocumented refugees. The camp refugees do not have freedom of movement and need to obtain special permission to go outside the camp. Anti-Rohingya sentiments prevail among the local Bangladeshi population, particularly owing to uneven competition in the local job market. Living conditions in the unregistered camps are destitute and often plagued by basic human rights violations. A representative example is summarized below:

“A pregnant woman aged 30 lived in the Rakhine state with her husband. In this region, Rohingya women often are forced to abort their fetus if they fail to disclose their pregnancy to the Nay-Sat Kut-kwey ye (NaSaKa) group and gain permission from them to have the baby. After getting apprised of the pregnancy, NaSaka soldiers burned down her house and slaughtered her husband with a sharp weapon. She said, ‘it felt like I got stuck in a horror story’. She wanted to save her child and live a life without this type of spine-tingling exploitations. Hence, her cousin put her in a fragile boat and led her to leave Myanmar. In the dark of night, they succeeded to deceive the coast guards and arrived in Bangladesh. She is now living in the shabby corner of an unregistered Kutupalong Makeshift Camp, which is known as a breeding zone for mosquitoes. During her pregnancy, to feed herself, she had to work 18 h a day with nominal wages. She gave birth to a baby girl, but was restrained to seek help from hospitals by the local police. Owing to the lack of proper treatment, her baby died after two days. She burst into tears while she was sharing her story. She told that ‘although I have no roof over my head and my heart turned blue due to the demise of my child, at least there is no-one here like the NaSaKa group, who can start vandalism and shatter my life in a moment. Sometimes it seems that there is no one in the world who can welcome us and treat our emergency’.”

## 4. Discussion

There has been a regular influx of Rohingya refugees into Bangladesh over the past century, owing to violent repression by Myanmar’s security forces, Buddhist extremism and discrimination against minority ethnic groups [3]. Most Rohingyas entering Bangladesh are granted temporary legal refugee status by the government, as a stopgap measure towards their eventual repatriation to Myanmar or resettlement to a third country (e.g., Australia, Canada, New Zealand, Sweden or United Kingdom). A steady stream of Rohingya continue to seek refuge in Bangladesh, despite the unfavorable conditions of unofficial refugee camps (which are considered to be preferable to the harsh reality faced in Myanmar).

UNHCR, along with other national and international agencies, are providing GOB with coordinated assistance for the refugees. In the two official Rohingya refugee camps there has been considerable improvement in the quality of shelter since 2006. Although the majority of huts have been rehabilitated, the space available per person is still inadequate, with limited privacy. There is a scarcity of potable water, which worsens during the dry season. The food basket that has been provided to the refugees at the registered camps since 2001 contains limited fresh goods, and lacks key vitamins and minerals. Each registered refugee receives around 2220 kcal of food each day, with 10.6 percent of this energy being provided by fat, which is less than the recommended 17 percent of energy [2]. Furthermore, following years of confinement under repressive camp conditions, mental health disorders continue to pose an ongoing concern and a challenge for humanitarian aid workers.

The position of GOB regarding Rohingya refugees in Bangladesh has focused more on providing transitory humanitarian relief rather than long-term integration into Bangladeshi society. There has been minimal action regarding improving living conditions, to deter influx of additional refugees. The repatriation of Rohingya refugees from Bangladesh to Myanmar has been hampered by the Myanmar government, which fails to address the Rohingya crisis and often perpetuates an environment of violence and persecution. While repatriation would be the preferred option of GOB for dealing with the Rohingya refugee crisis, the situation remains politically delicate.

GOB has adopted an increasingly reluctant stance towards the Rohingya issue. This has been attributed to two main reasons: (1) the government’s limited capacity, and (2) the complex Bangladesh-Myanmar border. A similar view was expressed during discussions with various stakeholders in Bangladesh. With a population of approximately 162.9 million [18], Bangladesh is one of the most densely populated countries in the world. The ongoing refugee influx creates a further strain on the poverty-stricken nation. Rohingya refugees are causing friction in the local community, as they are competing in the local job market and willing to work for less pay. They make up a significant portion of the local workforce in several industries, including construction, agriculture and salt production, and are involved in precarious jobs such as deep sea fishing. There is also concern about environmental degradation, for example by deforestation caused when they collect firewood for cooking. Additionally, there is an environment of instability in the nation, as some of the Rohingya refugees are involved with Islamic extremists and drug-traffickers. A few militant groups such as Rohingya Patriotic Front, Arakan Rohingya Islamic Front, Tehrek-Azadi Arakan and Rohingya Solidarity Organisation (RSO) are active at the border area of Myanmar and Bangladesh. These Islamic organizations are demanding a separate Islamic state are further complicating the Rohingya refugee crisis. Accordingly, GOB is cautious regarding the legal registration of Rohingya refugees entering Bangladesh.

## 5. Conclusions

GOB is facing mounting pressure from UNHCR and other international agencies to devise a more realistic strategy for managing the Rohingya refugee crisis. Dialogue between the governments of Myanmar and Bangladesh needs to be strengthened to jointly address the issue. However, as evidenced through Myanmar’s continued failure to address the problem, bilateral discourse is insufficient, and involvement of the global community has become imperative. It is the moral responsibility of the global community to address the plight of the Rohingya refugees by taking necessary measures to improve the situation of the Rohingya population within Myanmar. GOB may wish to enhance the screening of Rohingya refugees in terms of communicable diseases and criminal records. However, further assistance from private philanthropic organizations and international relief agencies will be needed to facilitate the timely humanitarian acceptance of this at-risk population into Bangladesh. Increasing levels of international aid will also help to curtail religious radicalization and criminal activity in this region. Through the combined efforts of the Myanmar government, GOB and international mediators—such as the Organisation of Islamic Countries (OIC) and United Nations—action can be taken towards a lasting solution to the ongoing Rohingya refugee crisis. Furthermore, culturally appropriate interventions are needed to address the debilitating mental health issues, such as chronic anxiety, grief, depression and posttraumatic stress, which are ubiquitous in this refugee population. Such future efforts, in tandem with appropriate political actions, will be key to finding a meaningful and sustained solution to this ongoing crisis.

## Figures and Tables

**Table 1:** Current information on refugees and undocumented persons from Myanmar currently in Bangladesh, RRRC, Bangladesh, 2015 [15].

Variables	Frequency (*n*)
Refugee camps	2
**Camp population (registered)**	
Kutupalong camp, Ukhiya	13,820
Nayapara camp, Teknaf	19,311
**Undocumented Myanmar citizen**	
Leda camp, Teknaf	15,000–20,000
Salampur, Teknaf	8000–10,000
Near Kutupalong camp (Tall), Ukhiya	40,000–50,000
Scattered in Cox’s Bazar and other districts	~300,000
**Basic support-providing institutions**	
Primary schools	21
Secondary schools	02
In-patient departments (IPD)	02
Out-patient departments (OPD)	02
**Food support through food card (E-voucher system)**	
Kutupalong (no. of families)	2627
Nayapara (no. of families)	3732

**Table 2:** Age and sex distribution of Myanmar refugees, RRRC, Bangladesh, 2015 [15].

Age Group (in Years)	Male	Female
	(*n* = 15,538)	(*n* = 17,592)
	(%)	(%)
0–4	6.5	7.2
5–17	19.7	20.0
18–59	18.3	25.4
≥60	1.3	1.2
Total	46.9	53.1

**Table 3:** Camp information on morbidity and mortality, UNHCR, 2015 [16,17].

Morbidity	Kutupalong	Nayapara
	(*n* = 13,102)	(*n* = 18,777)
	(%)	(%)
**Communicable Diseases**		
Upper respiratory tract infection	33.0	17.2
Lower respiratory tract infection	11.6	17.9
Diarrhea	7.1	9.0
Skin diseases	11.5	12.5
**Non-Communicable Diseases**		
Respiratory disorders	29.9	46.9
Endocrine and metabolic disorders	30.4	21.9
Cardiovascular disease	19.4	14.8
Others	16.4	20.3
**Mental Health**		
Epilepsy/seizures	26.7	57.1
Mental retardation/ intellectual disability	*	14.3
Psychotic disorders	43.3	*
Medically unexplained somatic complaints	*	9.5
Others	30.0	19.0
**Injuries**		
Accidents	89.5	64.3
Self-harm	*	18.4
Assault (no weapon)	5.3	12.5
Others	4.8	5.2
**Nutrition**		
Prevalence of global acute malnutrition (6–59 months)	13.0	13.0
Prevalence of stunting (6–59 months)	52.0	57.0
Prevalence of severe acute malnutrition (5–69 months)	2.0	1.0
Prevalence of anaemia (5–59 months)	43.0	49.0
Prevalence of anaemia in women of reproductive age	13.0	*
**Mortality**		
Crude mortality rate (CMR/1000/month	0.2	0.4
Under-five mortality rate (U5MR/1000/month)	0.4	1.0
Infant mortality rate (IMR/1000/livebirths)	19.6	45.4
Neonatal mortality rate (NNMR/1000/livebirths)	11.0	10.0

* Data not available.

**Table 4:** Access to healthcare, water and sanitation, UNHCR, 2015 [16,17].

Variables	Kutupalong	Nayapara
	(*n* = 13,102)	(*n* = 18,777)
Antenatal coverage (%)	94	92
Coverage to antenatal tetanus vaccination (%)	94	94
Proportion of births attended by skilled personnel (%)	100	98
Potable water available per person per day (L)	18	16
Refugees per latrine/toilet (*n*)	16	20
Proportion of households reporting defecating into a toilet (%)	95	90
Number of medical doctors (*n*)	4	4
Number of qualified nurses (*n*)	6	6
Number of community health workers (*n*)	16	19
